# PATTERNS OF HEAVY ALCOHOL USE AMONG OLDER MARRIED AND COHABITING COUPLES

**DOI:** 10.1093/geroni/igac059.2016

**Published:** 2022-12-20

**Authors:** Angela Curl, Jennifer Bulanda, Amy Roberts

**Affiliations:** Miami University, Oxford, Ohio, United States; Miami University, Oxford, Ohio, United States; Miami University, Oxford, Ohio, United States

## Abstract

Heavy drinking has increased among older adults in recent years. Research suggests romantic partners influence each other’s alcohol use, but this influence may vary by partnership type and relationship quality. Heavy drinkers are more likely to form cohabiting versus marital unions and, relative to marriage, cohabitation is linked with a higher risk of heavy episodic drinking. Although some studies have explored older married couples’ alcohol consumption, we lack research on older cohabitors despite the substantial growth in this form of partnership during later life. We use data from the 2014-2018 waves of the Health and Retirement Study to examine drinking behavior among a sample of older married and cohabiting couples (n=1,876) using Actor-Partner Interdependence Models (APIM). Heavy drinking is measured as consuming more than 3 drinks on a given day or 7 drinks per week for women, or 4 drinks on a given day or 14 per week for men (NIAAA, 2021). Compared to first marriages, being in a cohabiting union is associated with significantly higher odds of one (OR=5.22) or both (OR=3.82) partners becoming heavy drinkers over the observation period, but there are no significant differences between those in first marriages and remarriages. Relationship quality is also a significant correlate of heavy drinking risk, with male partners’ negative relationship quality and female partners’ positive relationship quality associated with a higher risk of heavy drinking over time. Results suggest the importance of accounting for partnership type and relationship quality in understanding partners’ drinking behavior.

